# Characteristics of Student-Led Clinics in the Allied Health Professions: Protocol for a Scoping Review

**DOI:** 10.2196/58084

**Published:** 2024-11-27

**Authors:** Sandra Robertson, Katie Thomson, Katrina Bannigan

**Affiliations:** 1 Glasgow Caledonian University Glasgow United Kingdom

**Keywords:** student-run clinic, student-facilitated clinic, allied health profession, interprofessional, higher education, university, tertiary education, preregistration, social care environment, practice based learning

## Abstract

**Background:**

Student-led clinics can provide students from allied health professions with the opportunity to gain valuable placement experience as an integral component of their preregistration program, enabling them to develop their competencies, professional skills, and administrative and leadership skills. Student-led clinics have the capacity to help meet the demand for appropriate practice-based learning opportunities, as there is an expectation that all allied health professions students should have high-quality learning experiences, ensuring the future workforce is fit for purpose. An overview of existing student-led clinics will increase our understanding of key characteristics, assisting education providers who may be considering the development of their own clinics. This will include key factors to ensure that this model of practice-based learning meets the needs of service users, students, and education providers.

**Objective:**

This scoping review aims to increase our understanding of the characteristics of student-led clinics by answering the questions (1) what student-led clinics exist in the allied health professions, and (2) what are their characteristics?

**Methods:**

This scoping review has been developed in conjunction with Joanna Briggs Institute methodology. We will consider studies and publications that include student-led clinics as an integral part of the preregistration curriculum for allied health professions students as defined by the Health and Care Professions Council. An extensive search of electronic databases will be conducted, including PubMed, MEDLINE, and CINAHL, among others. Search strategies, including the identified keywords and index terms, will be modified for each included database used. Reference lists of all included evidence will be screened for additional relevant studies. Studies published in English with no date limitations will be included. Relevant sources will be imported into Covidence for screening conducted by 2 reviewers (SR and KB). Data extraction will be conducted by 2 reviewers using a piloted data extraction tool, and data will be charted and tabulated using the Template for Intervention Description and Replication (TIDieR) checklist. Data will be presented with a narrative summary and illustrated by graphs and figures. The scoping review will be reported in conjunction with the PRISMA-ScR (Preferred Reporting Items for Systematic Reviews and Meta-analyses extension for scoping reviews) and the STORIES (Structured Approach to the Reporting In health care education of Evidence Synthesis) statement for health care education evidence synthesis.

**Results:**

An initial limited search was conducted in February 2024. The study will be conducted in 2025. Publication of the results is expected in late 2025.

**Conclusions:**

This scoping review will provide key information regarding the characteristics of student-led clinics and will be of interest to preregistration education programs within the allied health professions who have an interest in exploring opportunities to address placement capacity issues.

**International Registered Report Identifier (IRRID):**

PRR1-10.2196/58084

## Introduction

### Background

Practice-based learning is a core component of preregistration allied health programs and provides students with a valuable opportunity to develop the necessary skills and gain experience in health care delivery [1]. Meeting the demand for practice-based learning opportunities within the context of a changing health and social care environment is, however, challenging [1]. Increased student numbers and funding availability have been identified as areas leading to an increased demand for practice-based learning opportunities [2]. Strategies such as exploring different approaches to the traditional models of practice-based learning, for example, hospital-based placements, could help to address practice-based learning capacity issues [3].

There is an expectation that all allied health professions students should have high-quality, practice-based learning opportunities to ensure that the workforce is fit for the future, is able to develop effective partnerships, and have the skills to be able to flourish within a changing health and social care environment [4]. In the United Kingdom, the Health and Care Professions Council (HCPC) standards for preregistration education require practice-based learning to be an integral part of allied health programs and outline the responsibility of education providers to ensure the quality of practice-based learning [5]. The AHP Principles of Practice Based Learning highlight the requirement for students to engage in a diverse range of practice-based learning opportunities in order to reflect current health care provision, support the development of the professions, and shape the workforce of the future [6].

### Student-Led Clinics

Student-led clinics are a form of practice-based learning where students take responsibility for the organization and delivery of services under the supervision of staff [7]. They offer an innovative way of meeting demand in relation to providing opportunities for practice-based learning for allied health students, such as occupational therapy students. Student-led clinics are increasing as part of preregistration programs and provide experiential learning opportunities with a focus on clear goals and community services [8]. Student-led clinics present a valuable opportunity for students to participate in health care delivery [9]. The reasons for establishing student-led clinics internationally vary, but their focus could be on the support of underserved populations while addressing the challenges of meeting the demands of increasing student numbers and practice-based learning capacity issues [10]. Physiotherapy student-led clinics have demonstrated that they provide valuable educational opportunities for students while also providing cost-effective services to the public [11]. Student-led clinics can also provide valuable opportunities for interprofessional collaboration as they can be used to facilitate interprofessional learning experiences within a practice context [3]. In student-led clinics, students have the opportunity to develop their competencies and professional skills, as well as administrative and leadership skills, while also being able to take a lead role in the organization of the service being delivered under the supervision of a qualified health professional [7,12-14]. Other benefits of student-led clinics include the opportunity to interact with service users, work with students from other professions, learn about the role of other professions, and the development of clinical skills [7,15]. In addition, student-led clinics can enable students to develop their ability to demonstrate compassion and empathy for underserved populations and an opportunity to engage in experiences that may not normally be available as part of their education [9,16]. Reported benefits of student-led clinics include increased time for assessment and treatment, more holistic and integrated care, and increased access to services [17]. In contrast, the potential barriers to student-led clinics include sustainability from resource, time, and cost perspectives [7]. Other issues to be considered relate to the physical environment, such as space available and time commitments for practice educators [16]. This suggests that there are several factors to consider in the development of a student-led clinic, for example, its objectives, core features such as the patient population, the interprofessional care team, establishing community partnerships, and the use of technology [7,18]. Other factors to consider in the development of a student-led clinic are the structure of the clinic, its place within the curriculum [7], as well as engaging in a process of consultation with university stakeholders to explore the feasibility and accessibility of establishing a student-led clinic [19]. How the development of clinical skills would be measured, the training of educators, and the evaluation of outcomes for service users [7] would also be factors to consider in the development of a student-led clinic within an educational context.

However, limited research is available in relation to the characteristics of student-led clinics to guide their development in order to create effective educational opportunities for allied health students. As research related to student-led clinics is in its infancy [7], this review seeks to explore the characteristics of student-led clinics within the allied health professions in order to map and chart the existing evidence base, identify main concepts, and identify gaps in knowledge in this research area [20]. This will provide an in-depth understanding of the characteristics of student-led clinics within allied health professions, as the review will provide information relating to characteristics, such as types of clinics and students involved, aims of student-led clinics, populations served, and the place of the clinic within the curriculum [7]. This is with the aim of developing an effective solution to practice-based learning capacity issues as well as to inform future research. Exploring the existing evidence base in this area enables the development of student-led clinics to be based on current evidence and for valuable insight to be gained in relation to the purpose and characteristics of student-led clinics.

### Review Questions

This scoping review will focus on reviewing and collating data to provide an outline of the current body of research available in order to address the questions (1) what student-led clinics exist in the allied health professions, and (2) what are their characteristics?

## Methods

### Study Design

This review will be conducted in accordance with the Joanna Briggs Institute (JBI) methodology for scoping reviews to ensure a systematic methodology that can be replicated [21]. This scoping review is available as a project on the open science framework [22]. It will be reported in line with the PRISMA-ScR (Preferred Reporting Items for Systematic Reviews and Meta-analyses extension for scoping reviews) [20] and the STORIES (Structured Approach to the Reporting In health care education of Evidence Synthesis) statement for health care education evidence synthesis that provides specific guidance for reporting evidence synthesis within the context of health care education. This includes being specific about why a particular method of review was identified, how decisions were made in relation to the inclusion and exclusion criteria, and the process for data extraction [23].

### Eligibility Criteria

#### Participants

This review will focus on preregistration students from allied health professions. Preregistration allied health programs are approved educational programs that lead to eligibility to apply for registration with a regulatory body such as the HCPC within the United Kingdom. A preregistration program can be defined as a pathway into a profession [24]. The allied health professions included will be the 15 professions currently regulated by the HCPC in the United Kingdom. These include art therapists, biomedical scientists, podiatrists, chiropodists, clinical scientists, dietitians, hearing aid dispensers, occupational therapists, operating department practitioners, orthoptists, paramedics, physiotherapists, practitioner psychologists, prosthetists, orthotists, radiographers, and speech and language therapists [5]. In countries other than the United Kingdom, the term allied health may include different professions; however, for the purposes of this review, only allied professions as defined by the HCPC will be included.

Studies will be excluded if they focus on nursing, pharmacy, dentistry, or medical students, as they would not directly address the review question, which focuses on student-led clinics within allied health professions.

#### Concept

The concept being mapped within this scoping review will be the characteristics of student-led clinics, where students take responsibility for the organization and delivery of services under the supervision of staff [7]. This review will consider studies relating to student-led clinics with any type of service users and will not focus on a particular condition, diagnosis, or service user group. The drivers of student-led clinics can be to facilitate student skill development, provision of clinical placement hours, and social enterprise and altruism [7]. Student-led clinics that have been established with these or similar aims will be included in the review. Specific areas of interest are the characteristics of student-led clinics, such as type and location, duration, aims, type of allied health students, and development activities of the student-led clinic. Studies will be included that focus on a range of methods of delivery of student-led clinics, such as face-to-face, online, or hybrid delivery. As student-led clinics should be led by students [25], studies will be excluded where clinics are run by educators or practitioners, as these would potentially not provide insight into students in a leadership role. Studies will also be excluded whose primary focus is on aspects of student-led clinics, such as evaluating student and service user outcomes, cost-effectiveness, and satisfaction with service provision. Although these areas have been highlighted as being relevant for future research into student-led clinics [7], they are beyond the scope of this review. The Template for Intervention Description and Replication (TIDieR) will be used to extract relevant information from studies in order to present an accepted and methodical description of the characteristics of student-led clinics [26].

#### Context

The scoping review will consider studies that specifically focus on clinics, regardless of geographical location, that are led by allied health students within a higher education context. This is so that the student-led clinics included in the review will provide a learning experience for students as well as provide services [27]. The review will include studies that focus on student-led clinics that are an integral part of the preregistration curriculum [28]. Studies that focus on student-led clinics within a university context that are a formal part of the education of health professionals [9] will be considered within this scoping review; this will include studies that use student-led clinics at different stages within the curriculum [7]. Student-led clinics that take place in environments outside of a university-based setting (community and hospital settings) will be considered for inclusion as long as they are an integral part of the preregistration curriculum.

### Types of Sources

This scoping review will consider quantitative, qualitative, and mixed methods study designs for inclusion. Text and opinion papers will also be considered for inclusion in this scoping review. In addition, analytical observational studies including prospective and retrospective cohort studies, case-control studies, and analytical cross-sectional studies will be considered for inclusion. This review will also consider descriptive observational study designs including case series, individual case reports, and descriptive cross-sectional studies for inclusion. Qualitative studies will also be considered that focus on qualitative data including, but not limited to, designs such as phenomenology, grounded theory, ethnography, qualitative description, action research, and feminist research. In addition, reference lists of systematic reviews will be considered in order to identify potential studies.

### Review Team

The review is being conducted by a team comprised of experienced academics with an interest in occupational therapy practice-based learning (SR, KB, and KT).

### Patient and Public Involvement

This scoping review protocol includes the involvement of KB who is a practice educator and academic.

### Search Strategy

The search strategy aims to locate both published and unpublished studies. An initial limited search of MEDLINE, CINAHL, Cochrane Database of Systematic Reviews, PROSPERO (International Prospective Register of Systematic Reviews), and JBI Evidence Synthesis was conducted. There are 3 systematic reviews, a rapid evidence assessment, and a rapid review registered with PROSPERO with a focus on student-led clinics. The systematic review protocols identified focused on different aspects of student-led clinics, such as health interventions for adults with or at risk of chronic disease [29]; patient, client, consumer, and student outcomes of participating in a student-led clinic [30]; and colorectal cancer screening in free clinics [31]. The rapid evidence assessment focuses on the assessment of student-assisted assessment and brief intervention clinics to address gaps in remote and rural health care [32]. The rapid review focuses on the impact and scope of student-led clinics, but with a focus on rehabilitation science and student and service user outcomes [33]. In addition, a scoping review has been published in the last 3 years on the characteristics of student-led groups and clinics, but this relates specifically to physical rehabilitation [7]. As the primary focus of this proposed scoping review will be on the characteristics of student-led clinics, such as the type and location, duration, aims, type of allied health students, and development activities of any student-led clinic, it is not anticipated that these reviews will result in duplication as they either have a specific focus on student and service user outcomes, a particular condition, an aspect of rehabilitation, or an area of practice (remote and rural).

While conducting the initial limited search, the term “student led clinic” was mainly used. However, as studies were available both within the United Kingdom and in a global context, the terms “student run clinic” and “student facilitated clinic” were also included in recognition that different terminology may be used. During the initial search, the inclusion of the term “placement” in addition to search terms relating to student-led clinics, allied health students, and education limited the studies available. The search terms relating to education were therefore expanded to try and ensure that studies relating to student-led clinics as a model of placement were identified. Following the initial limited search, the text words contained in the titles and abstracts of relevant articles and the index terms used to describe the articles were used to develop a full search strategy for PubMed, MEDLINE, CINAHL, ERIC, Science Direct, PsycArticles, PsycINFO, TRIP database, and ProQuest Nursing and Allied Health Database, ProQuest One Academic, The Cochrane Library, and JBI Evidence-Based Practice database (Multimedia Appendix 1). The search strategy, which will include all the identified keywords and index terms, will be modified for each included database or information source. The reference list of all the included evidence sources will be screened for any relevant additional studies. Gray literature will also be searched, which will include the allied health professional body websites of the 15 professions currently regulated by the HCPC in the United Kingdom. This will include the Royal College of Occupational Therapists, the Chartered Society of Physiotherapists, and also the Royal College of Podiatry, among others. The professional bodies may also be contacted to source relevant materials relating to student-led clinics as a model of placement. Dissertations will also be searched through the ProQuest One Academic database, which provides access to ProQuest Dissertations and Theses Global. Studies published in English will be included, as the resources for translation are not available, and date limiters will not be used.

### Study or Source of Evidence Selection

Following the search, all identified citations will be collated and uploaded into a bibliographic software, or citation management system, and duplicates removed (ProQuest Ref Works). Potentially relevant sources will be retrieved and imported into the Covidence screening and data extraction tool. Following a pilot test, titles and abstracts will then be screened by 2 or more independent reviewers (SR and KB) for assessment against the inclusion criteria for the review. The full text of selected citations will be assessed in detail against the inclusion criteria by 2 independent reviewers (SR and KB). Reasons for the exclusion of sources of evidence in full text that do not meet the inclusion criteria will be recorded and reported in the scoping review. Any disagreements that arise between the reviewers at each stage of the selection process will be resolved through discussion, or with an additional reviewer (KT). The results of the search and the study inclusion process will be reported in full in the final scoping review and presented in a PRISMA-ScR flow diagram [20].

### Data Extraction

Data will be extracted from papers included in the scoping review by 2 independent reviewers using a data extraction tool based on the TIDieR checklist [23,26]. The TIDieR checklist was selected to aid the charting of clinical components, including participants, concepts, context, study methods, and key findings relevant to the review question.

A draft extraction form is provided in Multimedia Appendix 2, which will be piloted and then modified and revised as necessary. There will be 2 reviewers, 1 for extracting and 1 for checking (SR and KB). Initially, both reviewers will independently review a selection of studies for consistency in relation to what is being extracted. Any disagreements that arise between the reviewers will be resolved through discussion or with an additional reviewer. If appropriate, authors of papers will be contacted to request missing or additional data, as required. A logical and descriptive summary of the results that align with the questions of the review will be presented.

### Ethical Considerations

As the methodology of the scoping review consists of reviewing and collecting data from publicly accessible material, ethical approval is not required.

## Results

The initial scoping search conducted in February 2024 indicated that there was sufficient literature available to answer the research questions. The study will be conducted in 2025, with publication of the results expected in late 2025.

## Discussion

The scoping review will provide an outline of the current body of research relating to the characteristics of student-led clinics in the allied health professions that are an integral part of a preregistration curriculum. This scoping review will provide comprehensive information relating to the characteristics of student-led clinics across a range of allied health professions (as recognized by the HCPC), including arts therapists, biomedical scientists, podiatrists, chiropodists, clinical scientists, dietitians, hearing aid dispensers, occupational therapists, operating department practitioners, orthoptists, paramedics, physiotherapists, practitioner psychologists, prosthetists, orthotists, radiographers, and speech and language therapists [5]. The scoping review will also make an important contribution to gain a better understanding of how student-led clinics operate within preregistration programs.

This understanding of student-led clinics will contribute to the development of student-led clinics, gaining insight into their value in addressing placement capacity issues, and identifying gaps in knowledge in this research area and areas of future research [23].

It is important to highlight that there are some limitations in relation to conducting the proposed scoping review. While a comprehensive search strategy has been developed, there is a risk that some data that could have provided additional insights in relation to the characteristics of student-led clinics have been omitted. This risk is reduced by the process of searching gray literature and reference lists of included studies. As the aim of the scoping review is to identify and map evidence in a particular area [34], the studies included in the scoping review will not be critically appraised but will be used to develop insight and understanding in relation to the characteristics of student-led clinics. The reviewers therefore will be unable to comment on the quality of the studies that are included in the scoping review. In addition, the search strategy focuses on studies that have been published in English; therefore, some studies that would have been appropriate for inclusion, but published in other languages, could have been omitted from the scoping review.

The findings of the proposed scoping review will be of particular interest to preregistration education programs within the allied health professions, which have a focus on addressing placement capacity issues.

## Appendix

Multimedia Appendix 1 Search strategy.

Multimedia Appendix 2 Data extraction instrument.

## Abbreviations

HCPC: Health and Care Professions Council
JBI: Joanna Briggs Institute
PRISMA-ScR: Preferred Reporting Items for Systematic Reviews and Meta-analyses extension for scoping review
PROSPERO: International Prospective Register of Systematic Reviews
STORIES: Structured Approach to the Reporting In health care education of Evidence Synthesis
TIDieR: Template for Intervention Description and Replication

## Data Availability

All data that are relevant to the study are included within the article or uploaded as Multimedia Appendices 1-2.

## References

[1] McBride LJ, Fitzgerald C, Costello C, Perkins K (2020). Allied health pre-entry student clinical placement capacity: can it be sustained?. Aust Health Rev.

[2] Chambers M, Hickey G, Borghini G, McKeown R (2016). Preparation for practice: the role of the HCPC's standards of education and training in ensuring that newly qualified professionals are fit to practice. Health and Care Professions Council.

[3] Haines TP, Kent F, Keating JL (2014). Interprofessional student clinics: an economic evaluation of collaborative clinical placement education. J Interprof Care.

[4] (2023). AHP practice based learning (PrBL). NHS Education for Scotland.

[5] (2017). Standards of education and training. Health and Care Professions Council.

[6] (2023). AHP principles of practice based learning. Chartered Society of Physiotherapy.

[7] Wynne D, Cooper K (2021). Student-led rehabilitation groups and clinics in entry-level health education: a scoping review. JBI Evid Synth.

[8] Wynne D, Cooper K (2019). Student led physical rehabilitation groups and clinics in entry level health education: a scoping review protocol. JBI Database System Rev Implement Rep.

[9] Wilson OWA, Broman P, Tokolahi E, Andersen P, Brownie S (2023). Learning outcomes from participation in student-run health clinics: a systematic review. J Multidiscip Healthc.

[10] Forbes R (2020). Service dissatisfaction and non-attendance in physiotherapy student-led clinics: a qualitative study. Physiother Theory Pract.

[11] Forbes R, Nolan D (2018). Factors associated with patient-satisfaction in student-led physiotherapy clinics: a qualitative study. Physiother Theory Pract.

[12] Suen J, Attrill S, Thomas JM, Smale M, Delaney CL, Miller MD (2020). Effect of student-led health interventions on patient outcomes for those with cardiovascular disease or cardiovascular disease risk factors: a systematic review. BMC Cardiovasc Disord.

[13] Buckley E, Vu T, Remedios L (2014). The REACH project: implementing interprofessional practice at Australia's first student-led clinic. Educ Health (Abingdon).

[14] Broman P, Tokolahi E, Wilson OWA, Haggie M, Andersen P, Brownie S (2022). Patient outcomes from student-run health services: an integrative review. J Multidiscip Healthc.

[15] Sheu LC, Zheng P, Coelho AD, Lin LD, O'Sullivan PS, O'Brien BC, Yu AY, Lai CJ (2011). Learning through service: student perceptions on volunteering at interprofessional hepatitis B student-run clinics. J Cancer Educ.

[16] Kavannagh J, Kearns A, McGarry T (2015). The benefits and challenges of student-led clinics within an Irish context. JPTS.

[17] Stuhlmiller CM, Tolchard B (2015). Developing a student-led health and wellbeing clinic in an underserved community: collaborative learning, health outcomes and cost savings. BMC Nurs.

[18] McElfish PA, Hudson J, Schulz TK, Warmack TS, Moore R, Purvis RS (2017). Developing an interprofessional student-led clinic to address health disparities in a Pacific Islander Migrant Community. Journal of Student-Run Clinics.

[19] Pate LB, Dawson RM, Deupree J, Mitchell SM, Catledge CB (2022). A systematic approach to developing NP-led student health services clinic on a regional university campus. Nurs Forum.

[20] Tricco AC, Lillie E, Zarin W, O'Brien KK, Colquhoun H, Levac D, Moher D, Peters MDJ, Horsley T, Weeks L, Hempel S, Akl EA, Chang C, McGowan J, Stewart L, Hartling L, Aldcroft A, Wilson MG, Garritty C, Lewin S, Godfrey CM, Macdonald MT, Langlois EV, Soares-Weiser K, Moriarty J, Clifford T, Tunçalp Ö, Straus SE (2018). PRISMA extension for scoping reviews (PRISMA-ScR): checklist and explanation. Ann Intern Med.

[21] Peters MDJ, Godfrey C, McInerney P, Munn Z, Tricco AC, Khalil H, Aromataris E, Munn Z (2020). Scoping Reviews.

[22] Robertson S, Thomson K, Bannigan K (2024). Characteristics of student led clinics in the allied health professions: protocol for a scoping review. Open Science Framework.

[23] Gordon M, Gibbs T (2014). STORIES statement: publication standards for healthcare education evidence synthesis. BMC Med.

[24] (2019). Learning and development standards for pre-registration education. Royal College of Occupational Therapists.

[25] Simpson SA, Long JA (2007). Medical student-run health clinics: important contributors to patient care and medical education. J Gen Intern Med.

[26] Hoffmann TC, Glasziou PP, Boutron I, Milne R, Perera R, Moher D, Altman DG, Barbour V, Macdonald H, Johnston M, Lamb SE, Dixon-Woods M, McCulloch P, Wyatt JC, Chan A, Michie S (2014). Better reporting of interventions: template for intervention description and replication (TIDieR) checklist and guide. BMJ.

[27] Nagel DA, Naccarato TT, Philip MT, Ploszay VK, Winkler J, Sanchez-Ramirez DC, Penner JL (2022). Understanding student-run health initiatives in the context of community-based services: a concept analysis and proposed definitions. J Prim Care Community Health.

[28] Black JD, Palombaro KM, Dole RL (2013). Student experiences in creating and launching a student-led physical therapy pro bono clinic: a qualitative investigation. Phys Ther.

[29] Knowles R, Suen J, Miller M, Attrill S, Smale M, Thomas J (2019). Effectiveness of student-led health interventions in adults at risk or with chronic disease: a systematic review. PROSPERO.

[30] Charlton K, Morris S, Prideaux N, Smith M, Wadham D, Attrill S (2021). Outcomes from participating in student-led services or clinics in allied health practice: a systematic review. PROSPERO.

[31] Deshpande S, Tompkins W, Kamath S (2023). Colorectal cancer screening in free clinics: a systematic review. PROSPERO.

[32] Varela S, Stenhouse L, Subramaniam P, Wells G, Henderson M, Collins D (2021). Rapid evidence assessment of student-assisted assessment and brief intervention clinics: addressing the gaps in rural and remote health care. PROSPERO.

[33] Mollins J, Ashe M (2023). Exploring student-led clinics in rehabilitation science: a rapid review. PROSPERO.

[34] Munn Z, Peters MDJ, Stern C, Tufanaru C, McArthur A, Aromataris E (2018). Systematic review or scoping review? Guidance for authors when choosing between a systematic or scoping review approach. BMC Med Res Methodol.

