# Supplementary material for: Characteristics of Student-Led Clinics in the Allied Health Professions: Protocol for a Scoping Review
Source: JMIR Res Protoc. 2024 Nov 27;13:e58084. doi: 10.2196/58084 (PMC11635312; doi:10.2196/58084)
Supplement: Multimedia Appendix 1 [file resprot_v13i1e58084_app1.docx]

**Multimedia Appendix 1. Search strategy.**

| S24 | S7 AND S15 AND S22 | 203 |
| --- | --- | --- |
| S23 | S7 AND S15 AND S22 | 217 |
| S22 | S16 OR S17 OR S18 OR S19 OR S20 OR S21 | 175, 658 |
| S21 | (MH "Students, Allied Health") OR "student allied health" | 2, 170 |
| S20 | "third level education" | 24 |
| S19 | "tertiary education" | 817 |
| S18 | (MH "Colleges and Universities") OR "university" | 164, 126 |
| S17 | "higher education institute" | 50 |
| S16 | "higher education" | 12, 116 |
| S15 | S8 OR S9 OR S10 OR S11 OR S12 OR S13 OR S14 | 113, 482 |
| S14 | "professions supplementary to medicine" | 24 |
| S13 | "professions allied to medicine" | 80 |
| S12 | "health profession" | 57, 128 |
| S11 | "health occupation*" OR (MH "Health Occupations") | 6, 427 |
| S10 | "interprofessional" | 37, 819 |
| S9 | (MH "Allied Health Professions") OR "allied health profession" | 3, 756 |
| S8 | "allied health" | 22, 786 |
| S7 | S1 OR S2 OR S3 OR S4 OR S5 OR S6 | 3, 462 |
| S6 | (MH "Service Learning") OR "service learning*") | 1, 829 |
| S5 | "student group" | 814 |
| S4 | "student clinic" | 736 |
| S3 | "student facilitated clinic" | 0 |
| S2 | "student run clinic" | 77 |
| S1 | "student led clinic" | 45 |

Note: This search was run on CINAHL (via Ebsco) on 29 February, 2024.
