# Supplementary material for: Characteristics of Student-Led Clinics in the Allied Health Professions: Protocol for a Scoping Review
Source: JMIR Res Protoc. 2024 Nov 27;13:e58084. doi: 10.2196/58084 (PMC11635312; doi:10.2196/58084)
Supplement: Multimedia Appendix 2 [file resprot_v13i1e58084_app2.docx]

**Multimedia Appendix 2. Data extraction instrument (TIDieR) [1]**

| **Item Number** | **Item** | **Where located?** | |
| --- | --- | --- | --- |
|  |  | **Primary paper (page or appendix number)** | **Data Extracted** |
| 1 | **Brief name**  **(**Provide the name or a phrase that describes the intervention) |  |  |
| 2 | **Why**  (Rationale, theory, or goal of the elements essential to the intervention) |  |  |
| 3 | **What**  **Materials** (Describe any physical of informational materials used in the intervention and information on where the materials can be accessed) |  |  |
| 4 | **What**  **Procedures**(Describe each procedure, activity, and/or process used in the intervention) |  |  |
| 5 | **Who provided**  (For each provider, describe their expertise, background, and any specific training given) |  |  |
| 6 | **How**  (Describe modes of delivery) |  |  |
| 7 | **Where**  (Describe type(s) of location(s) where the intervention occurred) |  |  |
| 8 | **When and how much**  (Describe the number of times the intervention was delivered and over what period of time) |  |  |
| 9 | **Tailoring**  (If the intervention was planned to be personalised, titrated, or adapted, then describe what, why, when, and how) |  |  |
| 10 | **Modifications**  (Describe any changes during the course of the study/ intervention, any changing circumstances) |  |  |
| 11 | **How well**  **Planned**(If intervention adherence or fidelity was assessed, describe how and by whom; if any strategies were used to maintain or improve fidelity, describe them.) |  |  |
| 12 | **How well**  **Actual**(If intervention adherence or fidelity was assessed, describe the extent to which the intervention was delivered as planned.) |  |  |

1. Hoffman TC, Glasziou PP, Boutron I, Milne R, Perera R, Moher D, et al. Better reporting of interventions: template for intervention description and replication (TIDieR) checklist and guide. BMJ 2014 Mar 07; 348:g1687 DOI:10.1136/bmj.g1687
